# Antioxidant Effects of Some Drugs on Ethanol-induced Ulcers

**DOI:** 10.3390/molecules14020816

**Published:** 2009-02-18

**Authors:** Mira Popovic, Snezana Janicijevic-Hudomal, Biljana Kaurinovic, Julijana Rasic, Svetlana Trivic

**Affiliations:** 1 Department of Chemistry, Faculty of Science, University of Novi Sad, Trg Dositeja Obradovica 3, 21000 Novi Sad, Serbia; 2 Institute of Pharmacology, Faculty of Medicine, University of Pristina (Kosovska Mitrovica), Serbia

**Keywords:** Alcohol stress, Stomach, Bromocriptine, Haloperidol, Azithromycin, Antioxidative status.

## Abstract

The aim of this work was to investigate the antioxidant potential of some commonly used drugs (bromocriptine, haloperidol and azithromycin) on alcohol-induced ulcers in the rat. The following parameters were determined: content of reduced glutathione, activities of catalase, xanthine oxidase, glutathione reductase, glutathione peroxidase, peroxidase, and lipid peroxidation intensity. A battery of biochemical assays were used and the resulting data was statistically analyzed. Alcohol stress caused gastric ulcerations and hemorrhages and changed all the examined parameters except glutathione peroxidase activity. All drugs reduced the ulcer index and hemorrhages, with azithromycin showing the strongest effects. The drugs in combination with alcohol showed different effects on biochemical parameters. Our results indicate that the gastroprotective effects of the investigated drugs on experimental lesions induced by 100% ethanol could not be correlated with their antioxidative properties.

## Introduction

Oxidative stress plays an important role in the pathogenesis of more than 100 diseases [1]. Experimental studies have demonstrated that oxygen-generated free radicals (ROS) and lipid peroxidation are involved in the pathogenesis of acute gastric lesions induced by ethanol, nonsteroidal anti-inflammatory drugs or *Helicobacter pylori* [2,3,4,5].

Azithromycin (AZA) is an antibiotic with immunomodulatory effects which exhibits rapid and prolonged cellular accumulation, especially within phagocytes and shows anti-inflammatory action towards acute inflammation. Azithromycin also displays antioxidant effects [6]. Potential mechanisms of action include inhibition of cytokine release, neutrophyl function and mediator release, stimulation of apoptosis, as well as inhibition of mucus secretion [7,8,9]. 

Bromocriptine (BRC), with potent D2 receptor agonistic and mild D1 receptor antagonistic action, is widely used in the treatment of Parkinson’s disease and in a broad spectrum of psychiatric disorders [10]. Its mode of action is both central and peripheral and dose dependent. BRC has recently been shown to possess strong free radical scavenging action and antioxidant properties both *in vitro* and *in vivo* [11,12]. 

Haloperidol (HP) is a widely used neuroleptic drug used in the treatment of acute and chronic psychosis, e.g., schizophrenia. Haloperidol is thought to exert its clinical effects through cerebral dopamine D2-receptors and δ-opioid receptors [13]. Chronic treatment with neuroleptics increases free radical production and oxidative stress, and decreases the activity of antioxidant defense enzymes [14,15]. 

Numerous studies have shown that dopamine agonists such as bromocriptine, apomorphine L-Dopa and others, as well as dopamine antagonists such as haloperidol, domperidon, sulpirid etc., play a role in the modulation of stress ulcers, through dopamine receptors, both peripherally and centrally [16,17,18]. 

The commonly used medications bromocriptine, haloperidol and azythromycin have different chemical structures and different pharmacological actions, yet despite this they can all exert similar effects on the biochemical parameters examined in this work in the presence of alcohol. 

The aim of this work was to investigate the effects of bromocriptine, haloperidol and azithromycin on alcohol-induced ulcers, hemorrhages and antioxidative status of the stomach in experimental animals. For this purpose we measured catalase (CAT, E.C. 1.11.1.6), peroxidase (Px, E.C. 1.11.1.7), glutathione peroxidase (GSHPx, E.C. 1.11.1.9), glutathione reductase (GR. E.C. 1.6.4.2) and xanthine oxidase (XOD, E.C. 1.2.3.2) activities, reduced glutathione (GSH) content and lipid peroxidation (LPx) intensity. 

## Results and Discussion

In Table 1 we present the values of the measured parameters from stomachs of animals treated with alcohol in combination with bromocriptine, haloperidol and azithromycin. The GSH content was significantly reduced in all groups compared to the OO control group (untreated animals). A statistically significant decrease in GSH content was observed for the AH (alcohol + haloperidol, 25 mg/kg bw) and AB2 (alcohol + bromocriptine, 37.5 mg/kg bw) groups compared to the AO control (alcohol treated animals). 

Glutathione peroxidase activity was practically identical in both control groups, indicating that alcohol treatment did not change the activity of the enzyme. The glutathione peroxidase activity remained unchanged in all groups as compared to both controls (OO and AO). 

Alcohol-induced stress (AO group) increased the activity of glutathione reductase in a statistically significant manner. The activity of glutathione reductase was significantly reduced in all groups compared to the AO control group. In comparison to the OO group it was also significantly reduced in all groups except the AB3 (alcohol + bromocriptine, 25 mg/kg bw) and AH groups.

The peroxidase activity was significantly increased in all groups in comparison with the OO control. However, in all other groups no significant changes in Px activity were observed compared to the AO group. 

Activity of catalase (CAT) was significantly decreased in the following groups compared to the OO control: AO, AB2, AB3 and AH. On the other hand, a statistically significant increae of the CAT activity was recorded in the AB1 (alcohol + bromocriptine, 12.5 mg/kg bw), AB2, AB3 and AA (alcohol + azithromycin) groups, compared to the AO control group. 

**Table 1:** Investigated biochemical parameters.

Enzymes	OO group	AO group	AB1 group	AB2 group	AB3 group	AH group	AA group
GSH	1.55 ± 0.07	0.91 ± 0.08 ^c^	0.87 ± 0.03 ^c^	0.77 ± 0.05 ^c, d^	0.90 ± 0.04 ^c^	0.78± 0.03 ^c,e^	0.82 ± 0.05 ^c^
GSHPx	0.92 ± 0.10	0.91 ± 0.09	0.90 ± 0.07	0.89± 0.06	1.01± 0.13	1.06 ± 0.09	0.99 ± 0.11
GSHR	1.21 ± 0.04	1.42 ± 0.14 ^a^	0.99 ± 0.04 ^c,f^	0.80± 0.02 ^c,f^	1.17 ± 0.11 ^d^	1.19 ± 0.05 ^e^	1.08 ± 0.08 ^c, e^
Px	2.10 ± 0.07	2.93 ± 0.36 ^b^	2.46 ± 0.21 ^a^	2.48 ± 0.13 ^b^	3.40 ± 0.28 ^c^	3.11 ± 0.22 ^c^	3.26 ± 0.33 ^b^
CAT	2.11 ± 0.19	0.57 ± 0.14 ^c^	1.88 ± 0.17 ^f^	0.90 ± 0.18 ^c, d^	1.25 ± 0.12 ^c,f^	0.52 ± 0.12 ^ c^	2.47 ± 0.28 ^f^
XOD	3.25 ± 0.12	1.63 ± 0.21 ^c^	4.12 ± 0.16 ^ c, f^	4.55 ± 0.20 ^b,f^	3.66 ± 0.13 ^a,f^	5.11± 0.14 ^c, f^	4.74 ± 0.36 ^b, f^
LPx	0.23 ± 0.04	0.35 ± 0.09 ^c^	0.41± 0.02 ^c^	0.11± 0.01 ^b,e^	0.33 ± 0.02 ^c^	0.33 ± 0.03 ^b^	0.30 ± 0.03 ^a^
Ulcers index		16.20 ± 8.75	8.92 ± 4.79^d^	9.53 ± 4.22^d^	12.00 ± 5.92	4.50 ± 1.50^d^	0.00 ± 0.00^f^
Hemorrhages	stomach without change	pronounced submucosal hemorrhages	hemorrhages of lower intensity	hemorrhages similar to the control OO group	pronounced submucosal hemorrhages	hemorrhages present	significantly smaller change compared to the control, hemorrhages practically absent.

Activities of: XOD, CAT, PX, GSHPx and GSHR are expressed in nmol/mg of protein ·min^-1^

Intensity of lipid peroxidation is expressed in nmol malondialdehyde/mg protein

Content of GSH is expressed in nmol GSH/mg protein

n=5; compared to OO group: p>0.05 (statistically insignificant), ^a^ p<0.05, ^b^ p<0.01, ^c^ p<0.001; compared to AO group: p>0.05 (statistically insignificant), ^d^ p<0.05, ^e^ p<0.01, ^f^ p<0.001

Xanthine oxidase activity was statistically significantly reduced in the AO group, compared to the OO control, while it is statistically significantly increased in all other groups compared to both controls.

The lipid peroxidation (LPx) intensity was significantly increased in all groups, except AB2, compared to the OO control. In the AB group a statistically significant decrease was recorded compared to both control groups (OO and AO). 

Using one-way ANOVA (one-way analysis of variance) and Tukey Snedecor test F and D values, parameters which presents the total variability values and significance of differences observed between groups were assessed.

**Table 2:** ANOVA test for measured biochemical parameters.

GSH	00	A0	AB1	AB2	AB3	AH	GSHPx	00	A0	AB1	AB2	AB3	AH
**A0**	3.61 +						**A0**	0.01 -					
**AB1**	3.24 +	0.37 -					**AB1**	0.01 -	0.01 -				
**AB2**	2.18 +	1.44 +	1.07 +				**AB2**	0.02 -	0.01 -	0.01 -			
**AB3**	4.36 +	0.75 +	1.12 +	2.18 +			**AB3**	0.09 +	0.10 +	0.11 +	0.11 +		
**AH**	2.29 +	1.32 +	0.95 +	0.12 -	2.06 +		**AH**	0.14 +	0.14 +	0.15 +	0.16 +	0.04 -	
**AA**	2.68 +	0.93 +	0.56 +	0.51 +	1.68 +	0.39 -	**AA**	0.08 +	0.09 +	0.09 +	0.10 +	0.02 -	0.06 -
**GSHR**	00	A0	AB1	AB2	AB3	AH	**Px**	00	A0	AB1	AB2	AB3	AH
**A0**	12.07 +						**A0**	0.91 +					
**AB1**	7.78 +	4.30 +					**AB1**	0.46 +	0.45 +				
**AB2**	5.87 +	6.21 +	1.91 +				**AB2**	0.33 +	0.58 +	0.13 -			
**AB3**	13.58 +	1.51 +	5.81 +	7.72 +			**AB3**	0.50 +	1.41 +	0.96 +	0.83 +		
**AH**	9.63 +	2.44 +	1.86 +	3.77 +	3.95 +		**AH**	1.41 +	0.50 +	0.95 +	1.09 +	1.91 +	
**AA**	8.67 +	3.40 +	0.89 +	2.81 +	4.91 +	0.96 +	**AA**	2.98 +	2.07 +	2.52 +	2.65 +	3.48 +	1.57 +
**CAT**	00	A0	AB1	AB2	AB3	AH	**XOD**	00	A0	AB1	AB2	AB3	AH
**A0**	1.53 +						**A0**	1.82 +					
**AB1**	0.23 +	1.30 +					**AB1**	0.67 +	2.49 +				
**AB2**	1.36 +	0.17 +	1.13 +				**AB2**	0.90 +	0.92 +	1.57 +			
**AB3**	0.92 +	0.61 +	0.69 +	0.44 +			**AB3**	0.20 +	2.03 +	0.47 +	1.11 +		
**AH**	1.59 +	0.06 -	1.36 +	0.23 +	0.67 +		**AH**	1.65 +	3.48 +	0.98 +	2.56 +	1.45 +	
**AA**	0.37 +	1.90 +	0.60 +	1.73 +	1.28 +	1.95 +	**AA**	1.29 +	3.11 +	0.62 +	2.19 +	1.09 +	0.36 +
**LPx**	00	A0	AB1	AB2	AB3	AH							
**A0**	0.12 +												
**AB1**	0.17 +	0.06 +											
**AB2**	0.13 +	0.24 +	0.30 +										
**AB3**	0.01 -	0.12 +	0.18 +	0.12 +									
**AH**	0.09 +	0.02 -	0.08 +	0.22 +	0.10 +								
**AA**	0.07 +	0.04 +	0.10 +	0.20 +	0.08 +	0.02 -							

Results of the ANOVA test are represented for the differences between groups for the confidence level p<0.05; + statistically significant p < 0.05; - statistically nonsignificant p > 0.05; GSH: F= 26.99, p < 0.001; D = 0.40; GSHPx: F= 1.94, p > 0.05; D = 0.08; GSHR: F = 102.51; p < 0.001; D = 0.66; XOD: F = 140.52; p < 0.001; D = 0.17; CAT: F = 92.53; p < 0.001; D = 0.14; LPx: F = 21.64; p < 0.001; D=0.03; Px: F= 38.71; p < 0.001; D=0.31

GSH content was not significantly changed between the AO/AB1, AB2/AH and AH/AA groups. There was also no statistically significant difference between the following group combinations: AB3/OO, AH/AO and AA/AH in the case of LPx. Analysis of the activity of glutathione reductase (GSHR) and xanthine oxidase (XOD) pointed to statistically significant changes between all tested groups. CAT and Px activity showed that all group combinations have statistical significance differences except: AO/AH for CAT and AB1/AB2 for Px. GSHPx showed statistically insignificant differences in nine tested combinations. This further supports our experimental finding (Table 1) that there is no change in the activity of enzymes in all tested groups compared to the OO control. Based on results presented in Table 1 we can conclude that alcohol induced stress significantly affects the biochemical parameters of oxidative status investigated in this work with the exception of the activity of glutathione peroxidase. 

Compared to the AO group ulcer index and hemorrhages were significantly decreased in all groups, except the AB3 group (Table I). Alcohol-induced stress led to changes in the stomach indicated by profuse submucosal hemorrhages. Changes observed as erosions and petechiae, were confirmed pathohistologically as defects in mucosa and submucosal hemorrhages. In comparison with the AO group, the ulcer index was decreased by all tested drugs and the best protective effect was observed in the AA group. 

Results obtained in these experiments show a decrease in GSH content in animals treated with alcohol (AO group) compared to the control group (OO). GSH content could be decreased by its binding to acetaldehyde [19] due to elevated activity of gastric alcohol dehydrogenase (high ethanol concentration) leading to increased acetaldehyde concentration. It can also be decreased as a consequence of enzymatic or non-enzymatic GSH oxidation with ROS. Increased lipid peroxidation (LPx) intensity could also be one of the factors leading to the decrease in GSH content seen in this work. Thus, GSH could not exhibit its protective effects in animals, neither as an antioxidant nor in conjugation processes. Since GSHPx activity was not changed in comparison with the OO group, H_2_O_2_ probably could not affect GSH content. Furthermore, the activity of GSHR is significantly increased in the AO group alone, while in all combinations of the AO group with those treated with drugs it is reduced.

In the examination performed by La Casa *et al.* [20] it was established that ethanol causes a marked reduction in the mucosal non-protein SH content, expressed as GSH levels in rats. In addition, GSHPx activity decreases in gastric mucosa after ethanol treatment. Thiobarbituric acid-reactive substances in the gastric mucosa, used as an index of lipid peroxidation, increased markedly after administration of 50% ethanol.

Px activity was increased in all groups, especially in AB3, compared to OO group. High alcohol concentration leads to an NADH increase during ethanol conversion to acetaldehyde in the presence of gastric alcohol dehydrogenase. Hydrogen peroxide could be also produced by metabolization of ethanol and drugs. Increased NADH and hydrogen peroxide concentration could increase peroxidase activity, leading to decrease of substrate concentration for catalase. 

Activity of CAT was decreased in AO, AB2, AB3 and AH, and increased in the AA group compared to the OO group. The decrease in CAT levels leads to an increase in the accumulation of ROS, which can elevate the intensity of lipid peroxidation, tissue damage and increased peroxidase activity. 

Xanthine oxidase is a major source of ROS generation in the pathogenesis of various biological systems including the gastrointestinal tract [21]. XOD activity was decreased in AO group. In all other groups, particularly in AH group, it was increased, compared to both controls. Elevated XOD activity suggests that production of ROS (O_2_^- •^ and H_2_O_2_) is increased, what may lead to increased activity of antioxidant enzymes and LPx intensity. The effects of these drugs on alcohol-induced stress in the liver under the same experimental conditions gave similar values for the biochemical parameters studied in this work [22]. 

The highest ulcer index was observed in the AO group, while in the AA group it was similar to the control (OO). Furthermore, the ulcer index was recorded for all other groups, and values were found to be significantly lower, except in the AB3 group, compared to the AO group. Our results suggest that other processes besides lipid damage can lead to ulcerative injuries of mucosal membranes of the stomach, and that stress-induced ulcers are caused by an increase in free radical generation apart from acid pepsin factors. 

Naidu *et al*. [23] showed that chronic treatment of rats with HP significantly increases LPx intensity, and decreases GSH content and SOD and catalase activity. Our results are in accordance with these results, although we used the acute treatment in our investigation. 

Since bromocriptine effects are dose dependent, we applied three different doses. All of them had similar effects on the parameters investigated, probably due to bromocriptine scavenger activity [12]. The best gastroprotective effect was exerted azithromycin, which showed antioxidant effects as well [6]. 

Alcohol stress caused gastric ulcerations and hemorrhages and changed all of the examined biochemical parameters, except glutathione peroxidase activity. The effects of drugs on ethanol treated animals were variable. Sometimes they increased the analyzed parameters and sometimes they diminished it. However, the only direct measure of oxidative stress (i.e., TBARS levels) does not support the conclusion that drugs are gastroprotecive by an antioxidant mechanism. Only group AB2 had a significant decrease in TBARS. All other ethanol treated groups had values greater than control (OO).

All the tested drugs reduced the ulcer index and hemorrhage, with azithromycin showing the strongest effect. Importantly, AZA inhibits hemorrhaging completely and leaves ulcer index levels similar to control levels. Our results indicate that the gastroprotective effects of the investigated drugs on experimental lesions induced by 100% ethanol could not be correlated with their antioxidative properties. 

## Experimental

### General

This investigation was conducted on sexually mature males of laboratory Wistar rats, with an average body weight of 200-230 grams and ages up to 3 months. Rats were bred in the vivarium at the Centre for Biomedical Investigation, Galenika a.d. Animals were kept in standard Plexiglass cages at constant room temperature 22±1ºC, with circadian rhythm (day/night), and were fed standard laboratory rat feed, produced by the Veterinary Institute in Zemun. The number of rats was five per cage. Animals were treated according to the principles of the International Declaration Guide for Care and Use of Laboratory Animals (NIH publication № 85-23). Before the experiment, all animals were exposed to a 24-hour fasting period prior to treatment with alcohol, but had free access to water, and were put in metabolic plexiglass cages with a wire floor to prevent coprofagia. Average single doses of investigated drugs were selected on the basis of human dosage and Clark's formula. Experiments were conducted in the same day interval (8-15 h). Alcohol stress was induced by intragastric administration of 1 mL of 100% alcohol [24] Animals were returned to metabolic cages, but no water was given to them. After one hour, animals were sacrificed in ether narcosis. Animals that were not exposed to such stress and were not treated with any drugs were used as a control group.

The following drugs were used in the experiments: Bromocriptine^®^ (2.5 mg tablets, Zdravlje, Leskovac, Serbia), dissolved in distilled water immediatey prior to administration; Haldol^®^ decanoate (haloperidol decanoate, 50 mg/1 mL ampoule, Janssen-Cilag, Division of Johnson & Johnson S.E. d.o.o., Ljubljana, Slovenia); Hemomycin^®^ (azithromycin, 200 mg/5 mL, Hemofarm, Vrsac, Serbia). Of the drugs used in this study, only in the case of bromocriptine did we study the dose dependency since this drug has a wide therapeutic window in comparison with the other two. 

### Animal treatment

OO group- control – (untreated animals), AO group - alcohol (control without drugs), AB1 group - alcohol + bromocriptine, 24 h n.g. prior to stress 12.5 mg/kg bw. AB2 group – alcohol + bromocriptine n.g. 24 h prior to stress 37.5 mg/kg bw; AB3 group – alcohol + bromocriptine n.g. 3 h prior to stress 25 mg/kg bw; AH group – alcohol + haloperidol i.p. 30 minutes prior to stress 25 mg/kg bw; AA group – alcohol + azithromycin n.g. 250 mg/kg bw during 5 days prior to stress, on the fifth day 2.5 h prior to stress.

### Measurement of macroscopic changes in stomach epithelia following alcohol-induced stress

*Gastric lesion induction and evaluation*: Upon treatment, animals were sacrificed, and the abdomen was opened by midline incision, the stomach was removed, opened along the greater curvature, rinsed gently with water, and pinned open for macroscopic examination and for photodocumentation using a Canon Power Shot A40 digital camera. Areas with gastric lesions were measured by planimetry, using Get Area Lite for Corel Draw 11, and the ulcer index (UI) was estimated from the formula:

UI=[ulcerated area (mm^2^)/ total stomach area (mm^2^)]

Results are expressed as mean ± SD. Statistical analysis was done using the t-test. Differences with p<0.05 were considered to be significant. The ulcer index and the hemorrhaging area are the main indicators used in this work to estimate the anti-ulcer effects of the studied drugs. 

*Biochemical assays:* Animals were decapitated after treatment and the stomach was extracted. The stomach was homogenized in a Potter homogenizer with TRIS-HCl/sucrose in a ratio of 1:3 at 4^o^C. Obtained homogenates were filtered and the following biochemical parameters were determined: Extent of lipid peroxidation, LPx, was determined after Buege and Aust [25], peroxidase (Px) activity was measured after Simon *et al*. [26] and the effect of catalase (CAT) after Beers and Sizer [27]. Glutathione peroxidase (GSH-Px) activity was evaluated as described in Chin *et al*. [28], xanthine oxidase (XOD) after Bergmayer [29], glutathione reductase (GSHR) after Glatzle and Vuillenmir [30], and reduced glutathione content (GSH) after Kapetanović and Mieyal [31]. The total protein content was determined after Gornall *et al*. [32]

*Statistical analysis:* Results of biochemical analyses are presented as the mean value±standard deviation (S.D.). The difference between control and test groups was analyzed using the Student t-test (significant difference at p ≤ 0.05 confidence level). Using one-way ANOVA (one-way analysis of variance) and Tukey Snedecor test F and D values, parameters which presents the total variability values and significance of diferenecs observed between groups, were assessed. 
